# Novel clinical and dual infection by *Histoplasma capsulatum* genotypes in HIV patients from Northeastern, Brazil

**DOI:** 10.1038/s41598-019-48111-6

**Published:** 2019-08-13

**Authors:** Lisandra Serra Damasceno, Marcus de Melo Teixeira, Bridget Marie Barker, Marcos Abreu Almeida, Mauro de Medeiros Muniz, Cláudia Vera Pizzini, Jacó Ricarte Lima Mesquita, Gabriela Rodríguez-Arellanes, José Antonio Ramírez, Tania Vite-Garín, Terezinha do Menino Jesus Silva Leitão, Maria Lucia Taylor, Rodrigo Almeida-Paes, Rosely Maria Zancopé-Oliveira

**Affiliations:** 1 Hospital São José de Doenças Infecciosas – Secretaria de Saúde do Ceará, Fortaleza, Ceará Brazil; 2 Núcleo de Medicina Tropical, Faculdade de Medicina, Universidade de Brasília, Brasília Distrito Federal, Brazil; 3 Pathogen and Microbiome Institute, Northern Arizona University, Flagstaff, Arizona United States of America; 4 Instituto Nacional de Infectologia Evandro Chagas (INI), FIOCRUZ – Fundação Oswaldo Cruz, Laboratório de Micologia, 21045-900 Rio de Janeiro, RJ Brazil; 5 Facultad de Medicina, UNAM – Universidad Nacional Autónoma de México, Departamento de Microbiología y Parasitología, Laboratorio de Inmunología de Hongos, 04510 Ciudad de México, Mexico; 6 Faculdade de Medicina, UFC – Universidade Federal do Ceará, Departamento de Saúde Comunitária, 60430-140 Fortaleza, Ceará Brazil

**Keywords:** Fungal evolution, Fungal infection

## Abstract

Histoplasmosis is a worldwide-distributed deep mycosis that affects healthy and immunocompromised hosts. Severe and disseminated disease is especially common in HIV-infected patients. At least 11 phylogenetic species are recognized and the majority of diversity is found in Latin America. The northeastern region of Brazil has one of the highest HIV/AIDS prevalence in Latin America and Ceará State has one of the highest death rates due to histoplasmosis in the world, where the mortality rate varies between 33–42%. The phylogenetic distribution and population genetic structure of 51 clinical isolates from Northeast Brazil was studied. For that morphological characteristics, exoantigens profile, and fungal mating types were evaluated. The genotypes were deduced by a MSLT in order to define local population structure of this fungal pathogen. In addition, the relationships of *H*. *capsulatum* genotypes with clinically relevant phenotypes and clinical aspects were investigated. The results suggest two cryptic species, herein named population Northeast BR1 and population Northeast BR2. These populations are recombining, exhibit a high level of haplotype diversity, and contain different ratios of mating types *MAT1-1* and *MAT1-2*. However, differences in phenotypes or clinical aspects were not observed within these new cryptic species. A HIV patient can be co-infected by two or more genotypes from Northeast BR1 and/or Northeast BR2, which may have significant impact on disease progression due to the impaired immune response. We hypothesize that co-infections could be the result of multiple exposure events and may indicate higher risk of disseminated histoplasmosis, especially in HIV infected patients.

## Introduction

Histoplasmosis is a worldwide-distributed systemic mycosis caused by several cryptic species nested within the *Histoplasma capsulatum* complex^1^. *H*. *capsulatum* has a dimorphic life cycle; the mycelial phase (MP) develops in an environmental milieu with high concentrations of nitrogen and phosphorus, humidity above 60%, darkness, and near watercourses^2^. Upon inhalation of microconidia or macroconidia from *Histoplasma* spp. by susceptible hosts, the fungus differentiates into the yeast phase (YP), composed by budding yeast cells. Both MP and YP can be obtained by *in vitro* cultivation of the fungus at 25–28 °C and 34–37 °C, respectively. *Histoplasma* infections have been reported on all continents with exception of Antarctica^1^. The course of histoplasmosis varies from asymptomatic infection to mild-to-severe disease. High incidence of human *Histoplasma* infection has been reported mainly in tropical and subtropical areas of the Americas. However, the true disease range is likely larger, as this mycosis has also been found in Canada and in the Patagonia desert in Argentina^3,4^.

This pathogen can cause disease in different animal hosts, such as bats, domestic cats, and several wild mammals^2,4,5^. In humans, histoplasmosis outbreaks have been described in immunocompetent individuals linked to activities such as visiting caves and archeological sites, or working on a construction site. These exposures are often associated with environments containing high levels of bat or bird guano, which may favor the development of MP that harbors infectious microconidia^6^.

In last years, an increase of disseminated histoplasmosis has been reported, mainly associated with AIDS-patients throughout the Americas^7,8^. A major challenge is that mortality rate, presence of skin and mucosal lesions, and relapse frequency of these infections vary in individuals from distinct geographic areas of the disease^9,10^. In addition, experimental studies have demonstrated that variation in virulence may be associated with different genetic lineages of *H*. *capsulatum*^11^.

Multilocus sequence typing (MLST) is the main molecular tool currently used to evaluate genetic diversity at the species level of *H*. *capsulatum*^12^. The first analysis of isolates across the global distribution of the species defined 8 phylogenetic clades^13,14^. The LAm A and LAm B clades harbor isolates primarily from Latin America; the Nam 1 and Nam 2 clades from North America; the Eurasian clade from Egypt, India, China, Thailand, and England; the Netherlands clade; the Africa clade; and the Australian clade^14^. Recently, a more robust MLST study evaluating 234 isolates of *H*. *capsulatum* lead to the identification of at least 11 species-level clades, the majority of them found in Latin America. The former LAm A and LAm B species were divided into four different genetic clusters as follows: LAm A1, LAm A2, LAm B1 and LAm B2. Two new phylogenetic species, RJ (Southeast of Brazil) and BAC-1 (Mexico), and four different monophyletic and cryptic clades from Brazil (BR1-4) were also identified^1^.

Brazil presents one of the highest global incidences of histoplasmosis, and also presents the greatest genetic variability of *Histoplasma* and could be considered the center of origin of this important pathogen^1,15^. It is estimated that 2.19 individuals had a histoplasmosis diagnosis per 1,000 hospitalizations in Brazil^16^. However, the true incidence of this mycosis in Brazil is unknown, especially because it is not a notifiable disease.

Studies performed by histoplasmin skin-test between 1940 and 1990 have found different levels of prevalence of histoplasmosis in Brazil^17^. A prevalence rate of 93.2% was observed in southeastern Brazil^17,18^. In Ceará, a state located in the northeastern of Brazil, the prevalence rates varied from 23.6% to 61.5% among residents in rural areas^19,20^. Among HIV-positive individuals from Ceará without severe immunosuppression (lymphocyte T CD4+ >350 cells/mm^3^), the histoplasmosis prevalence reached 11.8%^21^. In the last three decades, the Ceará State has presented a large number of cases of disseminated histoplasmosis (DH) described mainly in AIDS-patients^9^. Between 1995 and 2004, 164 cases of co-infection of DH and AIDS were observed in a single hospital^22^. Moreover, 134 cases of the disease were found during a 7 year medical surveillance^23^; 208 cases in another 5 years^8^ and, more recently, 264 new cases in 7 years^9^ in this single state. These studies clearly demonstrate that northeastern Brazil, particularly Ceará State, is a highly endemic area of histoplasmosis, and is associated with a high annual death rate among HIV patients^24^. In Fortaleza (the capital of Ceará State), many cases of histoplasmosis are associated with low sanitation capacity, ecotourism, and fishing^25^.

Although antiretroviral therapy has modified the course of AIDS, a mortality rate between 33–42% associated with histoplasmosis has been observed in this region of Brazil^8,9^, unlike other endemic regions such as Panama (12.5%)^26^ and French Guiana (8%)^24,27^. The genetic background of *Histoplasma* in northeastern Brazil is poorly explored, especially considering the high mortality rates of DH so far reported for this particular area.

The aim of this study was to assemble the epidemiological, clinical, and laboratory data of histoplasmosis patients diagnosed in Ceará, Northeastern of Brazil. The phenotypes of clinical strains such as morphological characteristics, exoantigen profiles, and fungal mating types were evaluated. The genotypes of clinical isolates of *H*. *capsulatum* obtained from patients were deduced by a MSLT in order to define local population structure of this fungal pathogen. In addition, the relationships of *H*. *capsulatum* genotypes with clinically relevant phenotypes and clinical aspects were investigated. Finally, it was demonstrated the occurrence of recurrent infections by multiple genotypes of *H*. *capsulatum* in HIV patients.

## Results

### Clinical data

Relevant clinical data were evaluated in 43 hospitalizations of 40 individuals with DH from 2011 to 2014 (Table 1). Thirty-one cases occurred in males and nine in females. Patient’s age varied from 19 to 56 years old (median 31 years; interquartile range: 28–39 years). Only a single patient was HIV-negative. Hospitalizations were more frequent in individuals living in the metropolitan area of Fortaleza (75%). Other Ceará regions, such as the central wilderness, mountain region, and east coast, had smaller number of histoplasmosis cases (Table S1). All patients received amphotericin B deoxycholate (1 mg/Kg/day) until the clinical improvement, followed by itraconazole (400 mg/day). Co-infections with tuberculosis were detected in 15% of the cases. Fever, cough, and dyspnea were the most common clinical manifestations observed in the herein described patients (Table 1). Data revealed a high mortality ratio among the AIDS-patients (33%) included in this study. Moreover, presence of skin lesions (*p* = 0.003) and acute renal failure (*p* = 0.010) were risk factors associated with deaths.

**Table 1:** Baseline measurements of the histoplasmosis cases (n = 40) evaluated in the São José Hospital from Fortaleza, Ceará, Brazil, between 2011 to 2014.

Epidemiological data	
**Gender**	
Man	31 (77.5%)
Woman	9 (22.5%)
**Origin**	
Fortaleza, Ceará	23 (57.5%)
Other cities of Ceará	17 (42.5%)
**HIV/AIDS test**	
Positive	39 (97.5%)
Negative	1 (2.5%)
**Drug addictive**	
Yes	6 (15.0%)
No	34 (85.0%)
**Co-infection with tuberculosis**	
Positive	6 (15.0%)
Negative	34 (85.0%)
**Clinical characteristics**	
Fever	40 (100%)
Weight loss	33 (82.5%)
Cough	27 (67.5%)
Dyspnea	27 (67.5%)
Hepatomegaly	26 (65%)
Diarrhea	25 (62.5%)
Asthenia	22 (55%)
Spleenomegaly	20 (50%)
Vomit	18 (45%)
Abdominal pain	12 (30%)
Headache	11 (27.5%)
Mucosa hemorrhage	10 (25%)
Skin lesion	09 (22%)
Adenomegaly	08 (20%)
Acute renal failure	06 (15%)

### Macro and micromorphological characteristics of MP

Fifty-one clinical fungal isolates were obtained from the included patients. Fungi were isolated from buffy coat (n = 30), blood (n = 14), bone marrow (n = 6), and bronchoalveolar lavage (n = 1) (Table S1). The overall morphological characteristics of the *H*. *capsulatum* isolates are reported in Table S2, where differences in colony texture, color, and micromorphologies were recorded. The surface of *H*. *capsulatum* colonies varied from pale (white to beige) (36/51–70.6%) to dark (brown) (15/51–29.4%) and the texture of colonies ranged from cottony (42/51–82.4%) to powdery (9/51–17.6%). Microconidia were observed in all isolates while macroconidia were identified in 74.5% (38/51) of *H*. *capsulatum* isolates. There was no association between texture and presence of tuberculate macroconidia, as the majority of cottony (30/42–71.4%) and powdery colonies (8/9–89%) produced tuberculate macroconidia (*p* = 0.417).

### Dimorphism

The MP-YP conversion occurred in 88.2% (45/51) of *H*. *capsulatum* isolates in the first cultivation on ML-Gema Agar between 7 to 14 days. Five isolates (9.8%) presented a delayed MP-YP conversion, since the dimorphic switch took place after 3 or 4 sub-cultivations at the same conditions. Just one isolate (CE 0613) did not convert from MP to YP (2%) under the studied conditions (Table S2). All YP colonies presented smooth and moist texture, and showed characteristic oval-shaped yeast cells ranging from 2 to 5 µm in size.

### Exoantigen profiles

ID tests revealed that 17.6% of *H*. *capsulatum* exoantigens (9/51) yielded precipitin bands. Eight fungal isolates had single M band, and only one isolate had both H and M precipitin bands. By using Western blot, a more sensitive technique, the exoantigens were detected in all fungal isolates (Table S2). Both H and M antigens were observed in 29 (54.9%) isolates; 18 (35.2%) had single M band (corresponding to a  94 kDa protein), and 4 (7.9%) isolates just presented the H antigen (corresponding to a 114 kDa protein). There was no association between isolates that produced the both H and M antigens with texture (*p* = 0.268) or pigmentation (*p* = 0.167) of colonies, as well as with the presence or absence of macroconidia (*p* = 0.106).

### Phylogenetic distribution, population structure, and clinical/phenotype correlation

**Figure 1:**
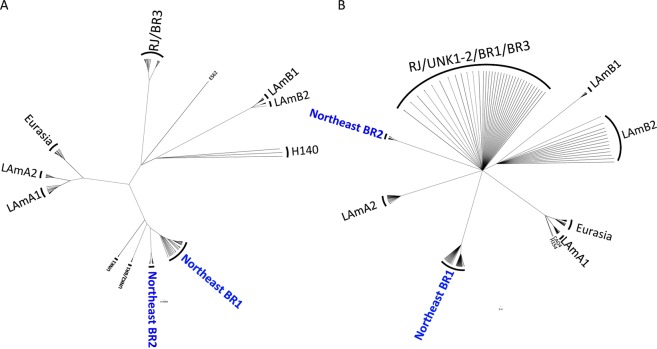
Phylogenetic distribution of the isolates from Northeast Brazil. Two cryptic clades Northeast BR1 and Northeast BR2 are revealed. Unrooted trees were generated by (**A**) Maximum Likelihood and (**B**) Bayesian analysis using K2P+ Inv Gamma DNA substitution model. Brach supports are proportion to the thickness of each branch.

Phylogenetic analysis revealed two new clades within Latin America, comprised mainly of the *H*. *capsulatum* isolates recovered from HIV-infected patients, named the Northeast clade BR1 and Northeast clade BR2 (Fig. 1). Both clades appear to be monophyletic using both maximum likelihood (ML) (Fig. 1A) and Bayesian inference (BI) methods (Fig. 1B). According to Teixeira *et al*.^1^, the strains from BR2 clade (84476, 84502, 84564, H151, and JIEF) and BR4 clade (H146, RE5646, and RE9463) nested within the herein proposed Northeast BR1 and Northeast BR2 populations, respectively. For that study, those clades were not classified as phylogenetic species due to the low taxon sampling (1). Therefore, the clades found in the Northeast region of Brazil were renamed in order to clarify nomenclature, Northeast clade BR1 and Northeast clade BR2. According to both ML and BI unrooted trees, the Northeast BR1 and BR2 clades are monophyletic; however, they have low bootstrap and posterior probability values (Fig. 1).

**Figure 2:**
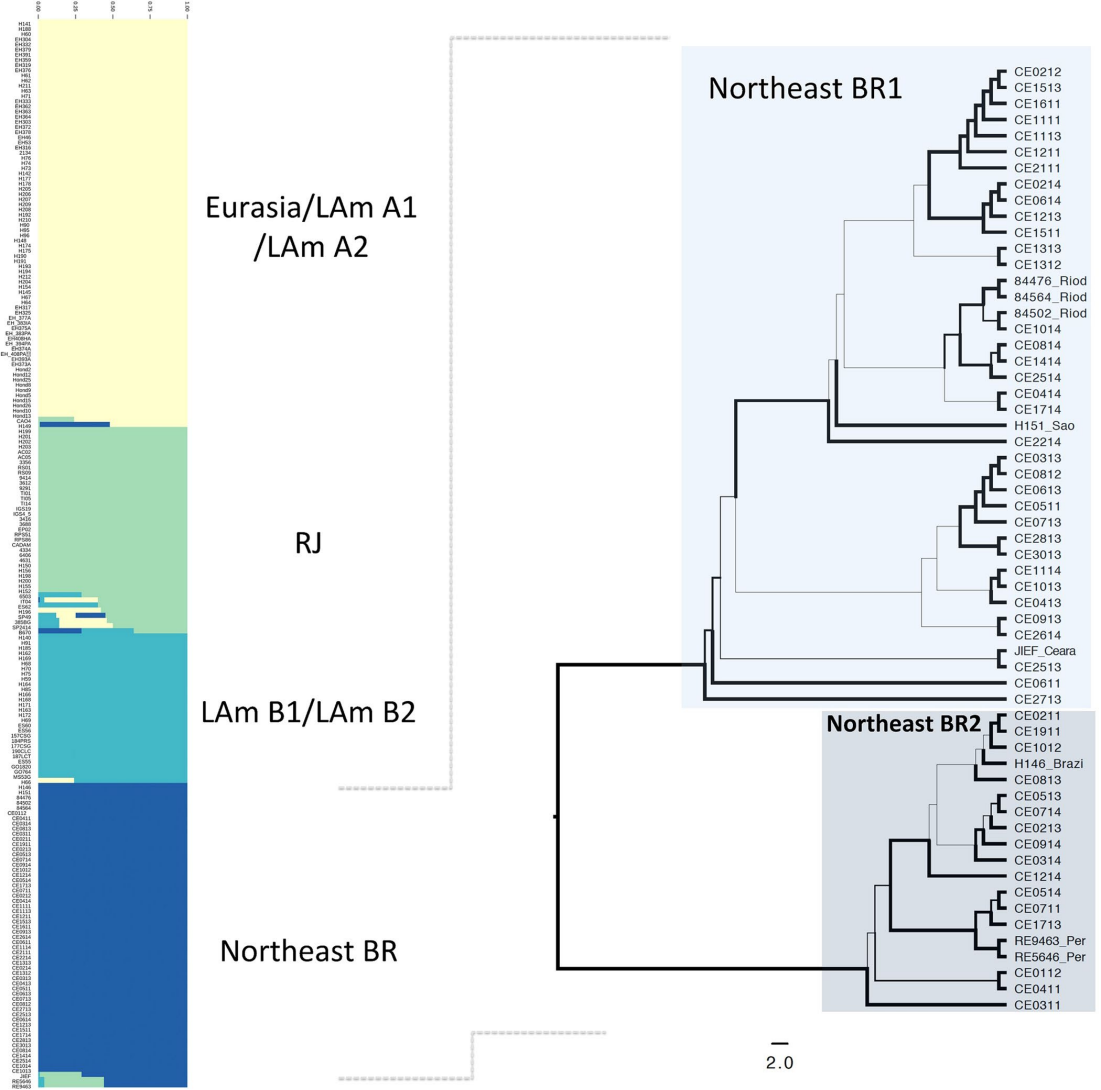
Population distribution of *H*. *capsulatum* in Latin America. (**A**) Admixture plots revealed a cryptic *H*. *capsulatum* population (green) harboring isolates from Northeast Brazil. Gene flow between Latin American populations is evidenced by admixed plots in RJ, LAm B and Northeast populations. (**B**) Phylogenetic analysis using the Maximum Likelihood methods of the Northeast population revealed two cryptic species Northeast1 and Northeast 2.

Initially, the whole population structure of Latin American (former LAm A, LAm B and Eurasia clades)^1^ was assessed via Bayesian Analysis of Population Structure using a Kmax value of 50. Initial admixture analysis revealed at least four clusters: Eurasia/LAm A1/LAm A2, RJ, LAm B1/LAm B2, and a fourth population composed mainly of isolates from the northeastern Brazil confirming the monophyly achieved in the phylogenetic analyses (Fig. 2). Gene flow between the RJ and Northeast BR1 and BR2 populations was noted, as the isolates JIEF, RE9463, and RE5646 share alleles from both populations (Fig. 2). Allele exchange has been extensively reported in the former LAm A clade, which is compatible with a sexually recombining species. Recombination analysis for the Northeast population was positive (p = 2.345 × 10^−12^) using the PHI-test analysis, and a cluster network analysis demonstrated gene flow between isolates within this population (Supplementary Fig. S1).

**Figure 3:**
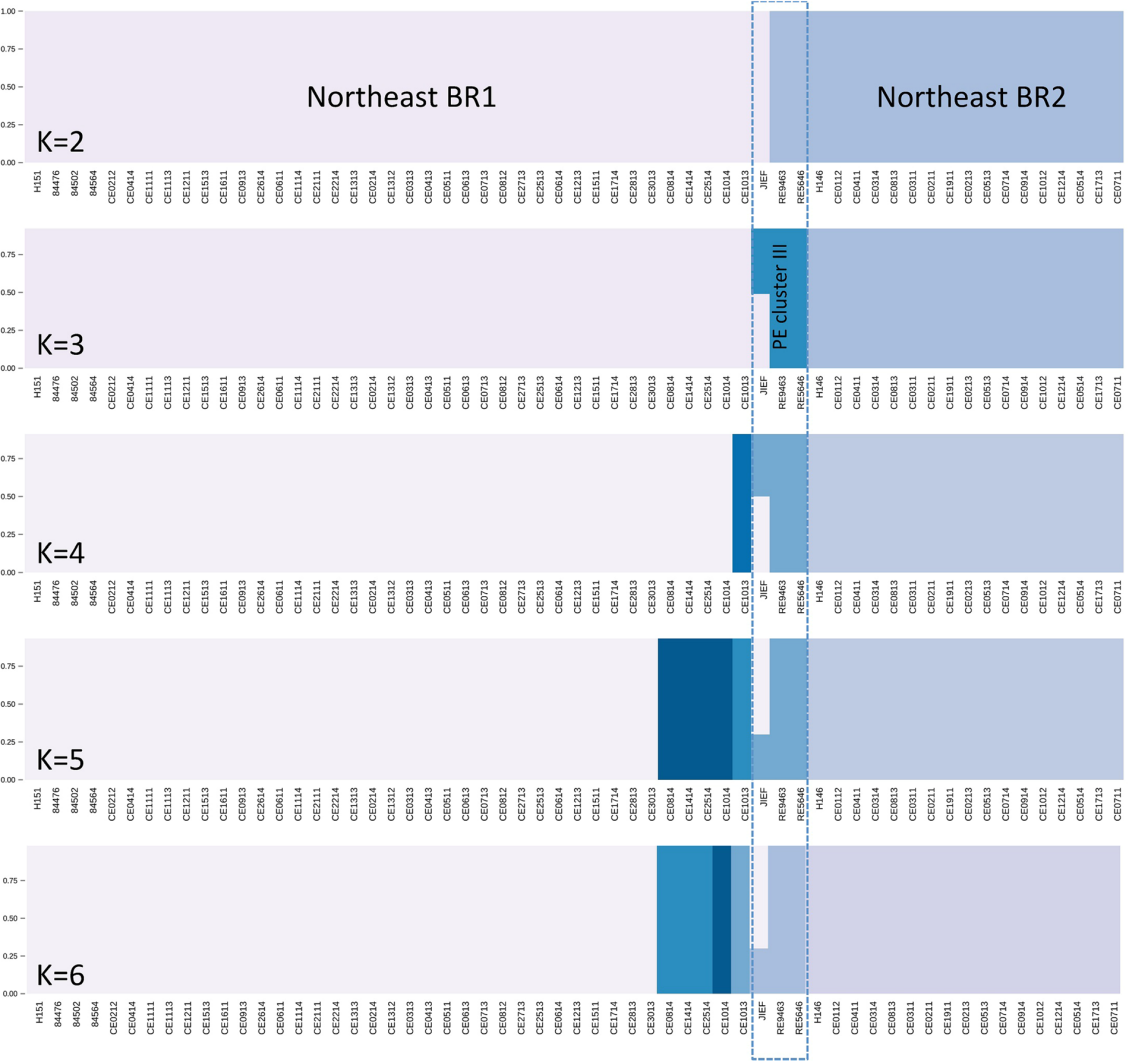
Sub-population distribution of Northeast isolates. Admixtures plots show the percentage of alleles unique or shares between Northeast BR1 and Northeast BR2 populations. At least three populations where found within Northeast isolates: The previous phylogenetic proposed Northeast BR1 and Northeast BR2 and an additional one composed by isolates of the neighbor state of Pernambuco, Brazil.

A deep investigation of the phylogenetic and population distribution of the 59 strains [51 from the present study and eight from Teixeira *et al*.^1^] belonging to the *Histoplasma* Northeast populations showed two monophyletic clades strongly supported by bootstrap values as represented by the ML tree (Fig. 2). Admixture analysis using a fixed K model revealed an optimal partition of K = 3: Northeast BR1, Northeast BR2, and a third population composed of two isolates (RE9463 and RE5646) from Pernambuco State, Brazil (a state that borders Ceará) and a third isolate (JIEF) from Ceará shares alleles with Northeast BR1 population and is likely a hybrid strain (Fig. 3).

**Figure 4:**
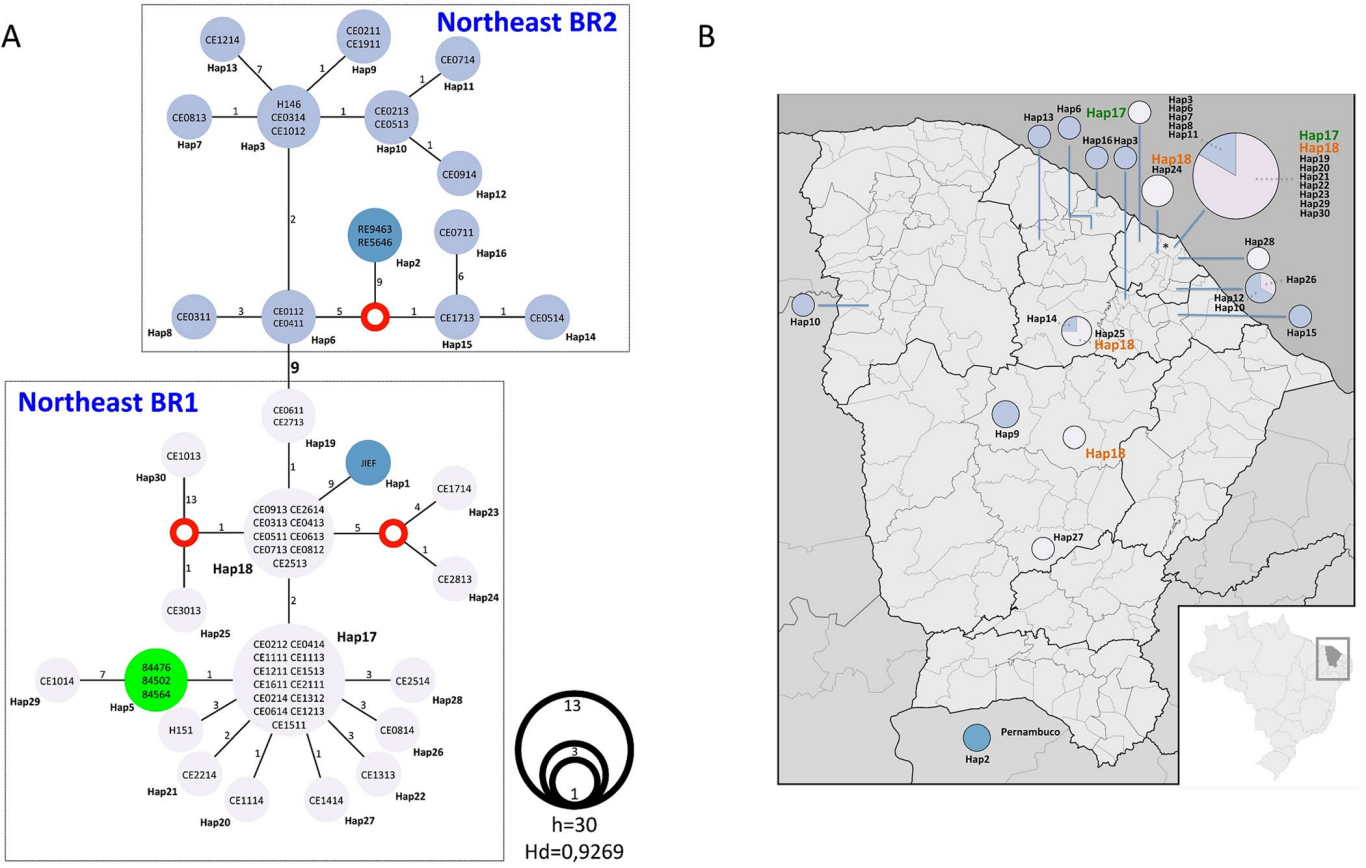
Haplotype network analysis using the Median-Joining method. (**A**) The haplotypes are proportional to the number of individuals and the number of mutations between each haplotype is displayed to each correspondent vertices. We identified 2 main population correspondent to Northeast BR1 and Northeast BR2 genotypes. (**B**) The haplotypes were plotted against each corresponding location in the map of Ceará state.

Haplotype networks revealed that the two Northeast populations are highly diverse (Fig. 4A). The haplotype diversity index for these populations is comparable to the highly recombinant RJ population (Hd = 0.9269)^1^. At least 30 haplotypes were observed within the subset of 59 isolates from northeastern Brazil (Fig. 4A). Northeast BR1 and Northeast BR2 are separated by nine mutations that are fixed in each population. The isolates RE9463 and RE5646 from Pernambuco form a unique haplotype (Hap2) derived from a median vector from a population Northeast BR2 (Fig. 4A). The isolates 84476, 84502, and 84564 were isolated in Rio de Janeiro in 1998 and represent cases diagnosed outside northeastern of Brazil belonging to Northeast BR1. Importantly, the population Northeast BR2 is widely spread across Ceará state while the Northeast BR1 is more restricted to the metropolitan area of the capital Fortaleza (Fig. 4B). This is evident as haplotypes 17 and 18 and their derivations (Hap19-23, Hap29, and Hap30) are clustered in Fortaleza and adjacent regions. Finally, it was observed that haplotype 18 is widely distributed in at least four municipalities and may represent a broadly distributed genotype (Fig. 4B). Associations between Northeast BR1 and Northeast BR2 populations and clinical manifestations such as dyspnea, mucosa hemorrhage, skin lesion, acute renal failure, and deaths were not observed (*p* > 0.05). Additionally, relationships between the genetic populations and their phenotypes were not detected either (Table 2, Supplementary Fig. S2). However, there was a slightly predominance of *MAT1-1* (68.7%) in Northeast BR2 and *MAT1-2* (62.9%) in Northeast BR1 (*p* = 0.036 – Table 2).

**Table 2:** Associations between phenotype and genotype of *H*. *capsulatum* isolates from Ceará, Brazil.

Phenotype	BR1 genotype	BR2 genotype	*p*-value
**Color**			
Pale	25 (71.4%)	11 (68.8%)	0.846
Dark	10 (28.6%)	5 (31.2%)	
**Texture**			
Cotony	30 (85.7%)	12 (75%)	0.352
Powdery	5 (14.3%)	4 (25%)	
**Micromorfology**			
Macroconidia+	26 (64.3%)	12 (75%)	0.957
Macroconidia−	9 (25.7%)	4 (25%)	
**Exoantigen**			
M antigen	15 (42.8%)	7 (43.8%)	0.952
M and H antigen	20 (57.2%)	9 (56.2%)	
**Mating**			
*MAT1-1*	13 (37.1%)	11 (68.8%)	**0**.**036**
*MAT1-2*	22 (62.9%)	5 (31.2%)	

### Dual *Histoplasma* infections in HIV patients

Seven patients included in this study yielded more than one *H*. *capsulatum* strain for analysis (Table S2). The phenotypic analyses of these strains revealed one patient (number 17) that yielded three isolates (CE 0313, CE 0713, CE 1013) with pale colonies, and one with dark pigmentation (CE 2713). The textures of *H*. *capsulatum* colonies obtained from two patients were cottony in some isolates (CE 0313, CE 0713, and CE 1013 from patient 17; CE 0513, and CE 0814 from patient 19) and powdery in others (CE 2713 from patient 17 and CE 0914 from patient 19). Tuberculate macroconidia were present in all isolates recovered from patients 2, 22 and 28; however, three patients (1, 17, and 19) were infected with fungal isolates with and without tuberculate macroconidia. The macroconidia were not observed on the isolates CE 0614 and CE 1014 from patient number 33. Moreover, the *H*. *capsulatum* isolates recovered from patients 2, 17, 19, 28, and 33 expressed different exoantigen profiles under the same experimental conditions.

The genotypic analyses revealed that sequential isolates belonging to the same haplotype were recovered from two patients (1 and 22). It is noteworthy that isolates CE0211 and CE1911 from patient 1, comprising the haplotype 9 within the Northeast BR2 population (Fig. 4), are phenotypically distinct, since CE1911 did not produce macroconidia. On the other hand, isolates CE1113 and CE1513 recovered from patient 22 are identical by means of genotypic (haplotype 17, Northeast BR1 population) and phenotypic analyses (Supplementary Fig. S2).

For the other five patients, more than one genotype was recovered from successive isolations (Table 3). Patients 2 and 19 were infected by isolates from the Northeast BR1 population (CE0511 and CE 0814, respectively) and from the Northeast BR2 population (CE0112 and CE0311 from patient 2; CE0513 and CE0914, from patient 19), while three patients (17, 28, and 33) were infected by different haplotypes within the Northeast BR1 population (Supplementary Fig. S3).

**Table 3:** Clinical and epidemiological features of patients infected with different genotypes of *Histoplasma capsulatum*.

Patients	Sex	Age	Risk activity	Symptoms	CD4+ (cells/mm^3^)	HAART at admission	Outcome
Patient 2	Man	22	no	Dyspnea	273	No	Discharged
Patient 17	Man	38	no	Dyspnea	Not performed	No	Discharged
Patient 19	Man	31	yes	Dyspnea	36	No	Discharged
Patient 28	Man	52	yes	Dyspnea, renal failure, skin lesions, mucosa bleeding	Not performed	No	Death
Patient 33*	Man	30	yes	Dyspnea	117	Poor adhesion	Discharged

^*^This individual was re-hospitalized with histoplasmosis and severe immunodeficiency (CD4+ 29 cells/mm^3^).

Of five patients affected with dual infection, all individuals were men, with average age of 34.6 years. Three patients had risk activity for histoplasmosis. Dyspnea was observed in all patients, and only one patient had skin lesions, mucosa bleeding and renal failure (patient 28). The first opportunistic infection in 4 among these 5 patients was histoplasmosis ou disseminated histoplasmosis was AIDS defining illness in 4 among these 5 patients. Only one patient had AIDS before this mycosis (patient 33), and this individual had irregular adhesion to HAART. One patient had tuberculosis co-infection (patient 2). Four patients had discharged and one died. Mortality of patients harboring different genotypes was similar to those infected with a single genotype (*p* = 1.000).

Three patients were re-hospitalized with the new hospitalization one to five months apart the first: patient 17 yielded four isolates with different phenotypic characteristics (Supplementary Figure 2) but all of them belonging to three distinct haplotypes from the Northeast BR1 population; patient 19 yielded three isolates from three different haplotypes, interestingly the two isolates from the first hospitalization (CE0513 and CE0914) were from the Northeast BR2 population and the isolate from the second hospitalization (CE0814) was from the Northeast BR1 population; finally patient 33 was infected by isolates with different haplotypes and mating types (CE0614 – Hap17, *MAT1-1*; CE1014 – Hap29, *MAT1-2*, from first and second hospitalizations, respectively), but both belonging to the Northeast BR1 population (Supplementary Figs S2 and S3).

## Discussion

Histoplasmosis has been considered an AIDS defining illness since 1987^28^, and a disease responsible for thousands of deaths in Latin America among people living with HIV/AIDS. The Ceará state, Northeastern Brazil, is considered the area with the highest mortality due to histoplasmosis in South America^8,9,24^, far surpassing rates in other endemic areas, such as Panama^26^ and French Guiana^24,27^. Thirty-one cases occurred in males and nine in females. These data has been previously demonstrated by Damasceno and collaborators (2013)^9^ where the majority of the subjects included in their studies were male with a mean age of 35 years (SD = 2.2; 95% CI = 3.01–3.75) and were born in the capital of Ceará. A lack of early diagnosis for the disease contributes to the high rates of mortality. The high mortality rate observed in this study, which unfortunately is expected for this disease among immunocompromised patients at this area, is in accordance with other studies conducted at Ceará, Brazil.

Currently, *H*. *capsulatum* is thought to be a complex of different species, containing at least 11 phylogenetic species and/or cryptic lineages^1^. Latin America presents higher diversity than other endemic areas, and diversity is highest in Brazil^1,29^. Additionally, the majority of *H*. *capsulatum* Brazilian clinical isolates previously studied were obtained from residents of southeastern Brazil^1,14^.

Fungal infections may contain multiple genotypes of the same pathogen, but still are usually considered as uniform entities. For the first time, we show strong support for two new populations, Northeast BR1 and Northeast BR2 that could cause mixed infections in HIV-positive individuals. The co-infection concept in virology is characterized by patients infected with two different virus strains simultaneously. On the other hand, the superinfection concept represents a condition in which an individual with established viral infection acquires a secondary infection provoked by a second genotype and this phenomenon is widely studied in viruses^30^. Similar event was observed in this research. Possibly, there were two different exposure events in the patients that presented different genotypes. Another explanation is that different genotypes share similar ecologic niches. Further studies are necessary to address these issues.

In bacteriology, this concept is slightly different; a secondary infection overcomes the earlier one caused by a different species and may be resistant to primary antibacterial treatment. Either way, mixed infections of *Mycobacterium tuberculosis* also have shown effects on both treatment and disease control^31^. Multiple infections due *Cryptococcus neoformans*^32^ and *Pneumocystis jirovecii*^33^ were also reported on HIV patients, however the incubation periods of fungal, bacterial and viral infections varies and whether the herein reported mixed infections are either co-infection or superinfections must be deeply revised in the medical mycology field.

Different genotypes may display divergent host cell tropism, immunologic evasion, or even antifungal-drug resistance, which is critical for patients with disseminated histoplasmosis and other endemic mycosis^34^. Thus, heterogeneous *H*. *capsulatum* infections may have significant consequences for immunologic escape and histoplasmosis progression. The competence of a previously cleared or ongoing *Histoplasma* infection to protect against a subsequent infection by a novel *Histoplasma* genotype may decrease the ability of the adaptive immune response to provide adequate and broad protection. The immunity induced by a prior *Histoplasma* infection could be insufficient to prevent a new infection. This directly challenges our current understanding of protective immunity, and impacts the development of vaccines for histoplasmosis and other endemic fungal pathogens in certain patient populations.

The genotyped isolates from Northeastern Brazil are contained in the previously delineated BR2 clade and BR4 clade respectively (Figs 1–4)^1^. The BR2 and BR4 clades were not previously ranked as phylogenetic species due to the low taxon sampling. However, we herein propose to elevate the status of “cryptic clades” to phylogenetic species of the former BR2 and BR4 (Fig. 1). The newly described *Histoplasma* Northeast BR1 and Northeast BR2 clades are monophyletic in both phylogenetic methods evaluated. However low bootstrap support for these monophyletic braches, likely due to homoplasy, were detected in both species so it is clear that more in depth analyses, such as whole genome sequencing, would resolve this question. Phylogenetic methods were coupled with population genetics analysis and it was identified the same population structure for the Northeast BR1 and Northeast BR2 clades mentioned above (Figs 2 and 3). Moreover, further investigation may reveal new populations in Northeast Brazil as evidenced by an isolated population of *H*. *capsulatum* in the Pernambuco state of Brazil (Fig. 3).

Additionally, this is the first study that evaluates the relationships between genotypes with clinical and phenotypic aspects of *H*. *capsulatum* isolates. It is known that DH can present with differential clinical characteristics in individuals from different endemic areas. For example, skin lesions and deaths are more frequent in individuals from Brazil than in patients from North America^10^. These differences may be related to different circulating genotypes on these two regions but also by phenotypic traits of the strains, such as different tolerances to cooler temperatures found in the skin. In fact the development and progression of histoplasmosis it is very sophisticated and depend of differences in fungal burden, disease kinetics, cytokine responses, and many virulence factors. Experimental studies have also found differences in the virulence of the pathogen, gene expression, and pathogenesis of disease between *H*. *capsulatum* from different phylogenetic clades^35–37^. Despite this, associations between genetic populations and phenotypic features were not detected (Table 2). Neither morphology nor exoantigen profile were associated with population/species, revealing that the phenotypic plasticity of those isolates is a process independent of speciation/population subdivision, and more likely due to intrinsic variation^38,39^.

Mixed mating types were identified with a slight predominance of one mating type in each group. Consequently, we hypothesize that both Northeast BR1 and Northeast BR2 are sexually recombining populations as evidenced by recombination analysis (phi-test), recombination networks, homoplasy, and admixture profiles (Figs 1, 3 and S1). Sexual reproduction can increase genetic diversity and may have consequences for pathogenesis by creating progeny with novel traits such as the ability to evade the host immune responses, a greater resistance to antifungal drugs, biofilm formation, or hyper virulence^40^. Thus, recombination events can facilitate shifts in lifestyles, which can persist in successive successful lineages^41^. In *Toxoplasma gondii*, mating crosses between type II and III virulent strains revealed that the recombinant f1 progeny had a virulence increased by 1,000-fold in a mice model^42^. More studies are necessary to characterize the impact of natural variation found within *H*. *capsulatum* populations and its influence on virulence and pathogenesis. Additionally, it was demonstrated that a single patient (patient #17) was infected by different mating types from the same phylogenetic species (Northeast BR1). We hypothesize that co-infections could be the result of multiple exposure events and may indicate higher risk of disseminated histoplasmosis, especially in HIV infected patients.

In summary, *H*. *capsulatum* clinical isolates from Ceará are genetically distinct from isolates from southern Brazil and Latin America, which demonstrates the high genetic diversity this pathogen. It is unclear how this relates to variation in phenotypes, and additional investigation is greatly needed in this area, such as a GWAS approach.

## Methods

### Histoplasmosis patients

The study was approved in the Research Ethical Committee of INI/FIOCRUZ (protocol number 19342513.2.0000.5262) and it is in accordance with the suggestions of the Ethics Committee of the School of Medicine, UNAM (protocol number 057–2014). Forty patients hospitalized at São José Hospital from Fortaleza, Ceará, Brazil, from 2011 to 2014 were included in this study. All patients had proven histoplasmosis by the isolation of *H*. *capsulatum* from clinical samples cultures. A retrospective study was conducted by reviewing medical records including epidemiological (sex, age, origin, occupational risk of histoplasmosis infection, drug addiction, and co-infection with tuberculosis), clinical (fever, weight loss, cough, dyspnea, hepatomegaly, diarrhea, asthenia, splenomegaly, vomit, abdominal pain, headache, mucosa hemorrhage, skin lesion, adenomegaly, and acute renal failure - ARF), and laboratory data (HIV serological test). Patient data entries were anonymously handled with Epi-Info software, version 7.1.5 (Centers for Disease Control and Prevention, Atlanta, GA, USA).

### Informed consent

Informed consent was obtained from all individual participants included in the study.

### Fungal isolates and DNA extraction

Fifty-one fungal isolates were obtained from the 40 patients seen at São José Hospital, located in Fortaleza (Ceará, Brazil), as mentioned above. *Histoplasma* colonies were isolated from diverse samples [blood, bone marrow, buffy coat and bronchoalveolar lavage (BAL)] by *in vitro* culture. YP or MP cells were grown in Ham’s F12 broth at 37 °C and 25 °C for 3–5 days, respectively. A total of 500 µl of YP/MP liquid cultures were used for DNA extraction. Briefly, the cells were harvested by centrifugation, washed three times with distilled deionized water, and kept at −4 °C for DNA extraction as previously described^43^. DNA was quantified by spectrophotometry using the Epoch™ Multi-Volume Spectrophotometer System (Biotek Instruments, Inc., USA).

### Phenotypic assays

Fungal isolates were maintained for 21 days on Potato Dextrose Agar plates (PDA – Difco, Detroit, MI, USA) at 25 °C in order to obtain the MP. Macromorphological features of *H*. *capsulatum* MP cultures, such as texture and color of colonies, were described. Micromorphological characteristics of MP were observed by optical microscopy (Zeiss PrimoStar, Oberkochen, Germany) by sampling 10 fields randomly with a magnification of 400X, after Lactophenol Cotton Blue (Fluka Analyted, France) staining. Dimorphism was demonstrated by MP-YP conversion in the ML-Gema Agar at 37 °C, for 7 to 14 days^43^.

Exoantigens were obtained from the 51 *H*. *capsulatum* isolates included in this study. Briefly, a fragment of 2–4 cm^2^ of *H*. *capsulatum* MP grown for 14 days on PDA slants was transferred to Erlenmeyer flasks containing 25 ml of Brain Heart Infusion broth (BHI - Difco, Detroit, MI, USA). Flasks were incubated at 25 °C in a gyratory shaker at 150 rpm for 7 days (New Brunswick Scientific, Edison, NJ). Thimerosal 1% was added in the seventh day to inactivate fungal cells, and the flasks were re-incubated overnight at 25 °C. Cultures were centrifuged at 1,050 × *g* for 10 min, and the supernatants were filtered through 0.45 µm pore size filter membranes (Nalgene Co., Rochester, NY). The pooled filtrate was concentrated to 50X in a Minicon Macrosolute B-15 Concentrator (Amicon Corp., Lexington, MA, USA)^44^. To evaluate the presence of the specific H and/or M antigens in the exoantigens from each *H*. *capsulatum* isolate, double immunodiffusion (ID) and western blot (WB) were performed. The ID assay was performed as previously described^13^. For the WB experiments, *H*. *capsulatum* exoantigens were initially separated by sodium dodecyl sulfate-polyacrylamide gel electrophoresis (SDS-PAGE), on 10% polyacrylamide resolving gels with a 4% polyacrylamide staking gel. The gels were then processed for WB according to the protocol previously established^45^ and revealed against a pool of sera from human patients with proven histoplasmosis.

### Mating type identification

The *MAT* locus of the *H*. *capsulatum* isolates was identified by a polymerase chain reaction (PCR) using previously described primers and reaction conditions^46,47^, where amplicons with 440 and 528 bp are expected after the amplification of *H*. *capsulatum MAT1-1* and *MAT1-2* loci, respectively. The G-217B (ATCC^®^ Number: MYA-2455^™^) from USA (*MAT1-1*) and G-186A (ATCC^®^ Number: 26029^TM^) from Panama (*MAT1-2*) reference strains were respectively used as controls for each mating type. Amplicons were resolved by 1.5% agarose gel electrophoresis. The 100-bp DNA ladder was used as a molecular size marker.

### Multi locus sequencing typing

Amplification of partial DNA sequences of four nuclear genes (*arf*, *H-anti*, *ole1*, and *tub1*) was performed according to the protocol described by Kasuga *et al*.^13^, with some modifications. The PCR reactions were achieved in a final volume of 25 µl, containing 200 µM of each deoxynucleoside triphosphate (dNTP) (Applied Biosystems Inc., Foster City, CA, USA), 2 mM of MgCl_2,_ 0.45 µM of each primer, 1.0 U of *Taq* DNA polymerase (New England BioLabs Inc., MA, USA), 1 X of *Taq* commercial buffer (New England BioLabs Inc., MA, USA) and 20 ng of each DNA template. The G-217B reference strain was used as positive control for the PCR reactions. PCR assays were performed in a Thermal iCycler (Bio-Rad Laboratories Inc., Hercules, CA, USA) programmed as follows: (a) 3 min at 95 °C; (b) 32 cycles, consisting of 15 sec at 94 °C, 30 sec at 65 °C in the first cycle, which was subsequently reduced by 0.7 °C/cycle for next 12 cycles, and 1 min at 72 °C. The remaining 20 cycles, the annealing temperature was continued at 56 °C; (c) a final extension cycle of 5 min at 72 °C (touchdown PCR)^48^. Generated amplicons were then sequenced by Sanger method at the High-Throughput Genomics Center (University of Washington) and the sequences were deposited in the GenBank database (http://www.ncbi.nlm.nih.gov) – (Table S3). The Asparagin Platform (http://asparagin.cenargen.embrapa.br/phph/) was used to analyze the electropherograms.

### Phylogenetic analysis

The obtained sequences were first checked by BLASTn^49^ to evaluate the genetic similarity with other *H*. *capsulatum* sequences deposited at GenBank. The sequences were aligned using the ClustalW^50^ algorithm implemented in the Mega 6.0 software^51^. We included the same dataset evaluated by Teixeira *et al*.^1^ to compare to the entire diversity of the genus *Histoplasma* so far reported (Table S3). The combined matrix was analyzed through two phylogenetic methods. First, maximum likelihood (ML) trees were generated using the IQ-TREE program^52^ using the –m MODEL function that allowing an automatic best-fit model selection (ModelFinder – K2P+ Inv Gamma was used in all tested phylogenies). The ultrafast bootstrap (UFBoot) approximation described by Minh *et al*.^53^ was employed to test branch confidence. Second, Bayesian inference (BI) was conducted using the MrBayes ver. 3.2 software^54^. Bayesian analysis was performed through 300,000 generations and samples were collected every 100 generations using 4 independent Markov Chain Monte Carlo (MCMC) to compute the posterior probability density. Twenty-five percent of the initial samples were discarded as *burn-in* and the remaining samples were used to build the consensual Bayesian tree. The consensus tree obtained by both methods was visualized in FigTree v1.3.1^55^.

### Recombination and population structure analysis

Recombination within Northeast populations was accessed using pairwise homoplasy index, (PHI-test). Cluster network analysis was inferred using the software SplitsTree 4^56^. Population distribution of Northeast *H*. *capsulatum* isolates was inferred using a Bayesian Analysis of Population Structure (BAPS)^57^. The haplotypic networks were inferred to visualize diversity both Northeast *H*. *capsulatum* populations. The distribution and diversity of haplotypes for the concatenated dataset was estimated using the software DnaSP, v 5^58^, and Median-joining networks were built and visualized in Network, v 4, software (Fluxus Technology, Clare, Suffolk, England). We conducted mixture and admixture analysis setting K-max to 50 hypothetical populations within the former LamA, LAmB as well as the Eurasian clade. In addition, fixed K model analysis (K = 2–5) was used for mixture analysis in order to infer the population sub-structuring within the Northeast population. In the admixture analysis (K = 3), 200 interactions were used to infer the admixture coefficient. Fifty references individuals were assumed for each cluster and admixture analyses were repeated 10 times per individual.

### Statistical analysis

The statistical analysis was carried out using the software STATA 11.2 (StataCorp LP, College Station, TX, USA). A bivariate analysis was performed to evaluate clinical data, phenotypic aspects, and genetic populations, using the Chi-square or Fisher exact test, if any value in the cells of the contingency table was less than five. A significance level of 5% (α = 0.05) was applied in all tests.

## Supplementary information

Dataset 1

## Data Availability

The datasets correspondent to the sequenced genes generated during the current study are available in the GenBank repository (https://www.ncbi.nlm.nih.gov/genbank/). Accession numbers and all other data generated or analysed during this study are included in this published articleand its Supplementary Information files.
