# Supplementary material for: Novel clinical and dual infection by Histoplasma capsulatum genotypes in HIV patients from Northeastern, Brazil
Source: Sci Rep. 2019 Aug 13;9:11789. doi: 10.1038/s41598-019-48111-6 (PMC6692370; doi:10.1038/s41598-019-48111-6)
Supplement: Supplementary file 1 — Dataset 1 [file 41598_2019_48111_MOESM1_ESM.pdf]

Supplementary Tables and Figures

Novel clinical and dual infection by *Histoplasma capsulatum* genotypes in HIV patients from Northeastern, Brazil

Lisandra Serra Damasceno**[,a], Marcus de Melo Teixeira**[b,c], Bridget Marie Barker[b,c], Marcos Abreu Almeida[d], Mauro de Medeiros Muniz[c], Cláudia Vera Pizzini[c], Jacó Ricarte Lima Mesquita[a], Gabriela Rodríguez-Arellanes[e], José Antonio Ramírez[e], Tania Vite-Garín[e], Terezinha do Menino Jesus Silva Leitão[,a,f], Maria Lucia Taylor***[,e], Rodrigo Almeida-Paes[d], Rosely Maria Zancopé-Oliveira***[,d]

[a]Hospital São José de Doenças Infecciosas – Secretaria de Saúde do Ceará, Fortaleza, Ceará

[b] Núcleo de Medicina Tropical, Faculdade de Medicina, Universidade de Brasília, Brasília, Distrito Federal

[c]Pathogen and Microbiome Institute, Northern Arizona University, Flagstaff, Arizona, United States of America

[d]Instituto Nacional de Infectologia Evandro Chagas (INI), FIOCRUZ – Fundação Oswaldo Cruz, Laboratório de Micologia, Setor Imunodiagnóstico, 21045-900 Rio de Janeiro, RJ, Brazil

[e]Facultad de Medicina, UNAM – Universidad Nacional Autónoma de México, Departamento de Microbiología y Parasitología, Laboratorio de Inmunología de Hongos, 04510 Ciudad de México, Mexico

[f]Faculdade de Medicina, UFC – Universidade Federal do Ceará, Departamento de Saúde Comunitária, 60430-140 Fortaleza, Ceará, Brazil

Corresponding author. lisainfecto@gmail.com Tel.: (+55 85) 31012320.

** These authors have equally involved in the the development of methodology, and data analyses.
***These authors have equally involved in the design, development, and funding of this study, and also contributed in the writing of this manuscript.

Table S1. Isolation and geographic informations of 51 *H.capsulatum* strains from Ceará, Brazil, 2011-2014.

Patients#	Isolate	Source	Date of culture	Location	Climate
1	CE 0211	Buffy coat	03/01/2011	Boa Viagem - Central wilderness of CE	Semi-arid
	CE 1911	Blood	02/02/2011	Boa Viagem - Central wilderness of CE	Semi-arid
2	CE 0311	Buffy coat	20/01/2011	Fortaleza - Metropolitan area of Fortaleza	Humid
	CE 0411	Bone marrow	20/01/2011	Fortaleza - Metropolitan area of Fortaleza	Humid
	CE 0511	Buffy coat	21/01/2011	Fortaleza - Metropolitan area of Fortaleza	Humid
3	CE 0611	Blood	29/12/2010	Fortaleza - Metropolitan area of Fortaleza	Humid
4	CE 0711	Buffy coat	18/01/2011	Paracuru - Metropolitan area of Fortaleza	Semi-arid
5	CE 1111	Bone marrow	17/12/2010	Caucaia - Metropolitan area of Fortaleza	Humid
6	CE 1211	Buffy coat	24/01/2011	Fortaleza - Metropolitan area of Fortaleza	Humid
7	CE 1511	Buffy coat	27/12/2010	Fortaleza - Metropolitan area of Fortaleza	Humid
8	CE 1611	Buffy coat	05/01/2011	Fortaleza - Metropolitan area of Fortaleza	Humid
9	CE 2111	Blood	10/02/2011	Fortaleza - Metropolitan area of Fortaleza	Humid
10	CE 0112	Blood	14/02/2012	São Gonçalo do Amarante - Metropolitan area of Fortaleza	Semi-arid
11	CE 0212	Buffy coat	10/04/2012	Fortaleza - Metropolitan area of Fortaleza	Humid
12	CE 0812	Buffy coat	05/07/2012	Quixeramobim - Central wilderness	Semi-arid
13	CE 1012	Buffy coat	21/08/2012	Fortaleza - Metropolitan area of Fortaleza	Humid
14	CE 1312	Buffy coat	16/11/2012	Fortaleza - Metropolitan area of Fortaleza	Humid

15	CE 0913	Blood	29/05/2013	Canindé - Central wilderness	Semi-arid
16	CE 0213	Buffy coat	11/04/2013	Guaraciaba do Norte - Moutain region of Ibiapaba	Humid
17	CE 0313	Buffy coat	16/04/2013	Fortaleza - Metropolitan area of Fortaleza	Humid
	CE 0713	Bone marrow	15/04/2013	Fortaleza - Metropolitan area of Fortaleza	Humid
	CE 1013	Buffy coat	17/04/2013	Fortaleza - Metropolitan area of Fortaleza	Humid
	*CE 2713	Buffy coat	13/09/2013	Fortaleza - Metropolitan area of Fortaleza	Humid
18	CE 0413	Blood	20/05/2013	Maracanaú - Metropolitan area of Fortaleza	Humid
19	CE 0513	Buffy coat	08/05/2013	Horizonte - Metropolitan area of Fortaleza	Humid
	CE 0914	Blood	14/05/2013	Horizonte - Metropolitan area of Fortaleza	Humid
	*CE 0814	Buffy coat	11/06/2013	Horizonte - Metropolitan area of Fortaleza	Humid
20	CE 0613	Buffy coat	11/04/2013	Fortaleza - Metropolitan area of Fortaleza	Humid
21	CE 0813	Bone marrow	25/02/2013	Fortaleza - Metropolitan area of Fortaleza	Humid
22	CE 1113	Bone marrow	09/04/2013	Fortaleza - Metropolitan area of Fortaleza	Humid
	CE 1513	Buffy coat	11/04/2013	Fortaleza - Metropolitan area of Fortaleza	Humid
23	CE 1213	Buffy coat	05/06/2013	Fortaleza - Metropolitan area of Fortaleza	Humid
24	CE 1313	Buffy coat	11/06/2013	Fortaleza - Metropolitan area of Fortaleza	Humid
25	CE 0414	Buffy coat	14/06/2013	Fortaleza - Metropolitan area of Fortaleza	Humid
26	CE 1414	Blood	10/05/2013	Acopiara - Central-south wilderness	Semi-arid
27	CE 1713	Buffy coat	18/01/2013	Chorozinho - Metropolitan area of Fortaleza	Humid
28	CE 2513	Buffy coat	27/08/2013	Maracanaú - Metropolitan area of Fortaleza	Humid

	CE 2813	Buffy coat	18/09/2013	Maracanaú - Metropolitan area of Fortaleza	Humid
29	CE 3013	Buffy coat	20/04/2013	Canindé - Central wilderness	Semi-arid
30	CE 0214	Blood	30/10/2013	Fortaleza - Metropolitan area of Fortaleza	Humid
31	CE 0314	Buffy coat	15/10/2012	Pacoti - Mountain region of Baturité	Humid
32	CE 0514	Buffy coat	15/07/2013	Canindé - Central wilderness	Semi-arid
33	CE 0614	Blood	31/05/2013	Fortaleza - Metropolitan area of Fortaleza	Humid
	*CE 1014	Bone marrow	11/10/2013	Fortaleza - Metropolitan area of Fortaleza	Humid
34	CE 0714	Buffy coat	05/06/2013	Fortaleza - Metropolitan area of Fortaleza	Humid
35	CE 1114	Blood	20/09/2013	Fortaleza - Metropolitan area of Fortaleza	Humid
36	CE 1214	Blood	30/10/2013	Itapajé - Eastern wilderness	Semi-arid
37	CE 1714	Blood	04/02/2014	Fortaleza - Metropolitan area of Fortaleza	Humid
38	CE 2214	Buffy coat	19/02/2014	Fortaleza - Metropolitan area of Fortaleza	Humid
39	CE 2514	BAL	23/01/2014	Euzébio - Metropolitan area of Fortaleza	Humid
40	CE 2614	Blood	01/02/2014	Fortaleza - Metropolitan area of Fortaleza	Humid

*Isolates obtained from re-hospitalized patients due a new episode of histoplasmosis

Table S2. Phenotypic and genotypic characteristics of *H. capsulatum* from Ceará, Brazil

#	Isolate	Color	Texture	Conidia	Dimorphism M-Y		Exoantigens		Mating type	Phylogenetic population	Haplotype
				**Macro/Micro**	**≤14 days**	**>14 days**	**ID**	**WB**			
1	CE 0211	Pale	Cottony	+/+	Yes	Yes	M	H/M	1-2	Northeast BR2	Hap9
	CE 1911	Pale	Cottony	-/+	Yes	Yes	-[!]	H/M	1-2	Northeast BR2	Hap9
2	CE 0311	Pale	Cottony	+/+	Yes	Yes	M	H/M	1-1	Northeast BR2	Hap8
	CE 0411	Pale	Cottony	+/+	Yes	Yes	-	H/M	1-1	Northeast BR2	Hap6
	CE 0511	Dark	Cottony	+/+	Yes	Yes	-	M	1-2	Northeast BR1	Hap18
3	CE 0611	Pale	Cottony	+/+	Yes	Yes	H/M	H/M	1-1	Northeast BR1	Hap19
4	CE 0711	Dark	Cottony	+/+	Yes	Yes	-	M	1-1	Northeast BR2	Hap16
5	CE 1111	Dark	Cottony	+/+	Yes	Yes	-	H/M	1-2	Northeast BR1	Hap17
6	CE 1211	Pale	Cottony	-/+	Yes	Yes	M	H/M	1-2	Northeast BR1	Hap17
7	CE 1511	Dark	Cottony	+/+	Yes	Yes	-	H	1-2	Northeast BR1	Hap17
8	CE 1611	Dark	Cottony	+/+	Yes	Yes	-	M	1-2	Northeast BR1	Hap17
9	CE 2111	Dark	Cottony	+/+	Yes	Yes	-	H	1-2	Northeast BR1	Hap17
10	CE 0112	Dark	Powdery	-/+	Yes	Yes	-	H	1-1	Northeast BR2	Hap17
11	CE 0212	Pale	Cottony	+/+	Yes	Yes	-	H/M	1-1	Northeast BR1	Hap17
12	CE 0812	Pale	Cottony	+/+	Yes	Yes	-	M	1-1	Northeast BR1	Hap18
13	CE 1012	Dark	Cottony	+/+	Yes	Yes	M	H/M	1-1	Northeast BR2	Hap3
14	CE 1312	Pale	Cottony	+/+	Yes	Yes	-	H/M	1-2	Northeast BR1	Hap17
15	CE 0913	Pale	Cottony	+/+	No	Yes	-	H/M	1-2	Northeast BR1	Hap18
16	CE 0213	Pale	Powdery	+/+	Yes	Yes	-	M	1-1	Northeast BR2	Hap10
17	CE 0713	Pale	Cottony	+/+	Yes	Yes	-	M	1-2	Northeast BR1	Hap18
	CE 0313	Pale	Cottony	+/+	No	Yes	-	H/M	1-2	Northeast BR1	Hap18
	CE 1013	Pale	Cottony	-/+	No	Yes	-	H/M	1-2	Northeast BR1	Hap30
	*CE 2713	Dark	Powdery	+/+	Yes	Yes	-	M	1-2	Northeast BR1	Hap19
18	CE 0413	Pale	Cottony	+/+	Yes	Yes	-	H/M	1-1	Northeast BR1	Hap18

	CE 0513	Pale	Cottony	+/+	Yes	Yes	M	M	1-1	Northeast BR2	Hap10
19	CE 0914	Dark	Powdery	+/+	Yes	Yes	-	M	1-1	Northeast BR2	Hap12
	*CE 0814	Pale	Cottony	-/+	Yes	Yes	-	H/M	1-2	Northeast BR1	Hap26
20	CE 0613	Pale	Cottony	+/+	No	No	-	M	1-1	Northeast BR1	Hap18
21	CE 0813	Pale	Cottony	+/+	Yes	Yes	-	H	1-2	Northeast BR2	Hap7
	CE 1113	Pale	Cottony	+/+	Yes	Yes	-	M	1-1	Northeast BR1	Hap17
22	CE 1513	Pale	Cottony	+/+	Yes	Yes	-	M	1-1	Northeast BR1	Hap17
23	CE 1213	Dark	Powdery	+/+	Yes	Yes	-	H/M	1-2	Northeast BR1	Hap17
24	CE 1313	Dark	Powdery	+/+	Yes	Yes	-	M	1-2	Northeast BR1	Hap22
25	CE 0414	Pale	Cottony	+/+	No	Yes	-	M	1-2	Northeast BR1	Hap17
26	CE 1414	Pale	Cottony	-/+	No	Yes	-	H/M	1-2	Northeast BR1	Hap27
27	CE1713	Dark	Powdery	+/+	Yes	Yes	-	H/M	1-2	Northeast BR2	Hap15
	CE 2513	Dark	Powdery	+/+	Yes	Yes	-	H/M	1-2	Northeast BR1	Hap18
28	CE 2813	Pale	Powdery	+/+	Yes	Yes	-	M	1-1	Northeast BR1	Hap24
29	CE 3013	Pale	Cottony	+/+	Yes	Yes	-	H/M	1-1	Northeast BR1	Hap25
30	CE 0214	Pale	Cottony	-/+	Yes	Yes	M	H/M	1-2	Northeast BR1	Hap17
31	CE 0314	Pale	Cottony	+/+	Yes	Yes	M	M	1-1	Northeast BR2	Hap3
32	CE 0514	Pale	Cottony	-/+	Yes	Yes	-	H/M	1-2	Northeast BR2	Hap14
	CE 0614	Pale	Cottony	-/+	Yes	Yes	-	H/M	1-1	Northeast BR1	Hap17
33	*CE 1014	Pale	Cottony	-/+	Yes	Yes	-	M	1-2	Northeast BR1	Hap29
34	CE 0714	Pale	Cottony	-/+	Yes	Yes	-	H/M	1-1	Northeast BR2	Hap11
35	CE 1114	Pale	Cottony	-/+	Yes	Yes	-	H/M	1-1	Northeast BR1	Hap20
36	CE 1214	Pale	Cottony	+/+	Yes	Yes	-	H/M	1-1	Northeast BR2	Hap13
37	CE 1714	Dark	Cottony	+/+	Yes	Yes	M	H/M	1-2	Northeast BR1	Hap23
38	CE 2214	Pale	Cottony	-/+	Yes	Yes	-	H/M	1-2	Northeast BR1	Hap21
39	CE 2514	Pale	Cottony	+/+	Yes	Yes	-	M	1-2	Northeast BR1	Hap28
40	CE 2614	Pale	Cottony	+/+	Yes	Yes	-	H/M	1-1	Northeast BR1	Hap18

#Patient number / *Isolates obtained from re-hospitalized patients due a new episode of histoplasmosis / (+) present (-) absent / !Negative

Table S3: Dataset of *H. capsulatum* strain/isolate used in the phylogenetic and admixture analysis.

**Isolate**	**State/Country**	**arf**	**H-anti**	**ole1**	**tub1**
CE0112	Ceará/Brazil	KX756766	KX756853	KX756900	KX756945
CE0211	Ceará/Brazil	KX756787	KX756852	KX756899	KX756946
CE0212	Ceará/Brazil	KX756788	KX756851	KX756898	KX756903
CE0213	Ceará/Brazil	KX756789	KX756850	KX756897	KX756944
CE0214	Ceará/Brazil	KX756767	KX756855	KX756896	KX756918
CE0313	Ceará/Brazil	KX756768	KX756849	KX756895	KX756904
CE0314	Ceará/Brazil	KX756769	KX756848	KX756894	KX756943
CE0413	Ceará/Brazil	KX756770	KX756847	KX756893	KX756905
CE0414	Ceará/Brazil	KX756783	KX756846	KX756892	KX756906
CE0513	Ceará/Brazil	KX756784	KX756845	KX756891	KX756935
CE0514	Ceará/Brazil	KX756785	KX756814	KX756890	KX756939
CE0611	Ceará/Brazil	KX756786	KX756844	KX756889	KX756914
CE0613	Ceará/Brazil	KX756771	KX756843	KX756888	KX756907
CE0614	Ceará/Brazil	KX756772	KX756842	KX756887	KX756917
CE0711	Ceará/Brazil	KX756765	KX756813	KX756886	KX756940
CE0713	Ceará/Brazil	KX756773	KX756841	KX756885	KX756908
CE0714	Ceará/Brazil	KX756790	KX756840	KX756884	KX756941
CE0812	Ceará/Brazil	KX756774	KX756839	KX756883	KX756909
CE0813	Ceará/Brazil	KX756775	KX756838	KX756882	KX756936
CE0814	Ceará/Brazil	KX756791	KX756837	KX756881	KX756916
CE0913	Ceará/Brazil	KX756792	KX756836	KX756880	KX756910
CE0914	Ceará/Brazil	KX756793	KX756835	KX756879	KX756942
CE1012	Ceará/Brazil	KX756794	KX756834	KX756878	KX756937
CE1013	Ceará/Brazil	KX756776	KX756856	KX756877	KX756921
CE1014	Ceará/Brazil	KX756795	KX756833	KX756876	KX756919
CE1111	Ceará/Brazil	KX756796	KX756832	KX756875	KX756920
CE1113	Ceará/Brazil	KX756797	KX756831	KX756874	KX756911
CE1114	Ceará/Brazil	KX756798	KX756830	KX756873	KX756922
CE1211	Ceará/Brazil	KX756799	KX756829	KX756901	KX756923

CE1213	Ceará/Brazil	KX756777	KX756828	KX756872	KX756926
CE1214	Ceará/Brazil	KX756778	KX756827	KX756871	KX756947
CE1312	Ceará/Brazil	KX756779	KX756854	KX756870	KX756924
CE1313	Ceará/Brazil	KX756800	KX756826	KX756869	KX756925
CE1414	Ceará/Brazil	KX756801	KX756825	KX756868	KX756927
CE1511	Ceará/Brazil	KX756780	KX756824	KX756867	KX756915
CE1513	Ceará/Brazil	KX756802	KX756823	KX756866	KX756912
CE1611	Ceará/Brazil	KX756803	KX756822	KX756865	KX756929
CE1713	Ceará/Brazil	KX756804	KX756812	KX756902	KX756938
CE1714	Ceará/Brazil	KX756781	KX756821	KX756864	KX756930
CE1911	Ceará/Brazil	KX756805	KX756820	KX756863	KX756948
CE2111	Ceará/Brazil	KX756806	KX756819	KX756862	KX756913
CE2214	Ceará/Brazil	KX756807	KX756818	KX756861	KX756931
CE2514	Ceará/Brazil	KX756808	KX756817	KX756860	KX756934
CE2614	Ceará/Brazil	KX756809	KX756816	KX756859	KX756932
CE2713	Ceará/Brazil	KX756782	KX756815	KX756858	KX756933
CE3013	Ceará/Brazil	KX756810	KX756811	KX756857	KX756928
CE0311	Ceará/Brazil	KX058302	KX058322	KX058307	KX058312
CE0411	Ceará/Brazil	KX058301	KX058321	KX058306	KX058311
CE0511	Ceará/Brazil	KX058300	KX058320	KX058305	KX058310
CE2813	Ceará/Brazil	KX058298	KX058318	KX058303	KX058309
CE2513	Ceará/Brazil	KX058299	KX058319	KX058304	KX058308
2761	Alabama/USA	JX443639.1	JX458497.1	NI	NI
385BG	MS/Brazil	GU320865.1	GU320903.1	GU320993.1	GU321043.1
H64	Bogota/Colombia	Tree base #1063	Tree base #1063	Tree base #1063	Tree base #1063
H67	Medellin/Colombia	Tree base #1063	Tree base #1063	Tree base #1063	Tree base #1063
H145	Surinam	Tree base #1063	Tree base #1063	Tree base #1063	Tree base #1063
Hond10	Honduras	JQ218428.1	JQ218367.1	JQ218408.1	JQ218382.1
Hond13	Honduras	JQ218431.1	JQ218370.1	JQ218411.1	JQ218396.1
Hond18	Honduras	NI	JQ218374.1	JQ218415.1	JQ218397.1
Hond19	Honduras	NI	NI	JQ218416.1	NI

SP2414	SP/Brazil	GU320867.1	GU320901.1	GU320995.1	GU321045.1
EH317	Morelos/Mexico	Tree base #1063	Tree base #1063	Tree base #1063	Tree base #1063
EH325	Chiapas/Mexico	Tree base #1063	Tree base #1063	Tree base #1063	Tree base #1063
Hond3	Honduras	JQ218422.1	JQ218362.1	JQ218401.1	JQ218393.1
Hond7	Honduras	JQ218425.1	NI	JQ218405.1	JQ218381.1
Hond9	Honduras	JQ218427.1	JQ218366.1	JQ218407.1	JQ218395.1
Hond11	Honduras	JQ218429.1	JQ218368.1	JQ218409.1	NI
H149	Sao Paulo/Brazil	Tree base #1063	Tree base #1063	Tree base #1063	Tree base #1063
SP49	SP/Brazil	GU320866.1	GU320902.1	GU320994.1	GU321044.1
84476	Rio de Janeiro/Brazil	GU320841.1	GU320890.1	GU321008.1	GU321084.1
84502	Rio de Janeiro/Brazil	GU320840.1	GU320888.1	GU321006.1	GU321068.1
84564	Rio de Janeiro/Brazil	GU320842.1	GU320889.1	GU321007.1	GU321085.1
H151	Sao Paulo/Brazil	Tree base #1063	Tree base #1063	Tree base #1063	Tree base #1063
JIEF	CE/Brazil	GU320862.1	GU320906.1	GU320990.1	GU321040.1
ES62	Espirito Santo/Brazil	GU320871.1	GU320897.1	GU320999.1	GU321049.1
H154	Sao Paulo/Brazil	Tree base #1063	Tree base #1063	Tree base #1063	Tree base #1063
H196	Rio de Janeiro/Brazil	Tree base #1063	Tree base #1063	Tree base #1063	Tree base #1063
H146	Brazil	Tree base #1063	Tree base #1063	Tree base #1063	Tree base #1063
RE5646	PE/Brazil	GU320860.1	GU320908.1	GU320988.1	GU321038.1
RE9463	PE/Brazil	GU320861.1	GU320907.1	GU320989.1	GU321039.1
H90	Egypt/Africa	Tree base #1063	Tree base #1063	Tree base #1063	Tree base #1063
H95	Egypt/Africa	Tree base #1063	Tree base #1063	Tree base #1063	Tree base #1063
H96	India	Tree base #1063	Tree base #1063	Tree base #1063	Tree base #1063
H142	England	Tree base #1063	Tree base #1063	Tree base #1063	Tree base #1063
H148	NI	Tree base #1063	Tree base #1063	Tree base #1063	Tree base #1063
H174	Poland	Tree base #1063	Tree base #1063	Tree base #1063	Tree base #1063
H175	Poland	Tree base #1063	Tree base #1063	Tree base #1063	Tree base #1063
H177	Beijing/China	Tree base #1063	Tree base #1063	Tree base #1063	Tree base #1063
H178	Beijing/China	Tree base #1063	Tree base #1063	Tree base #1063	Tree base #1063
H190	NI	Tree base #1063	Tree base #1063	Tree base #1063	Tree base #1063
H191	NI	Tree base #1063	Tree base #1063	Tree base #1063	Tree base #1063

H192	India	Tree base #1063	Tree base #1063	Tree base #1063	Tree base #1063
H193	Egypt	Tree base #1063	Tree base #1063	Tree base #1063	Tree base #1063
H194	Egypt	Tree base #1063	Tree base #1063	Tree base #1063	Tree base #1063
H204	India	Tree base #1063	Tree base #1063	Tree base #1063	Tree base #1063
H205	Thailand	Tree base #1063	Tree base #1063	Tree base #1063	Tree base #1063
H206	Thailand	Tree base #1063	Tree base #1063	Tree base #1063	Tree base #1063
H207	Thailand	Tree base #1063	Tree base #1063	Tree base #1063	Tree base #1063
H208	Thailand	Tree base #1063	Tree base #1063	Tree base #1063	Tree base #1063
H209	Thailand	Tree base #1063	Tree base #1063	Tree base #1063	Tree base #1063
H210	Thailand	Tree base #1063	Tree base #1063	Tree base #1063	Tree base #1063
H212	Algeria	Tree base #1063	Tree base #1063	Tree base #1063	Tree base #1063
Hond1	Honduras	NI	JQ218360.**1**	JQ218399.**1**	JQ218379.**1**
Hond2	Honduras	JQ218421.1	JQ218361.1	JQ218400.1	JQ218386.1
Hond8	Honduras	JQ218426.1	JQ218365.1	JQ218406.1	JQ218388.1
Hond12	Honduras	JQ218430.1	JQ218369.1	JQ218410.1	JQ218389.1
Hond14	Honduras	JQ218432.1	JQ218371.1	JQ218412.1	NI
Hond25	Honduras	JQ218435.1	JQ218377.1	JQ218419.1	JQ218385.1
Meles	Germany	JX093565.1	JX093566.1	NI	JX093567.1
H91	Guinea-Liberian Border/Africa	Tree base #1063	Tree base #1063	Tree base #1063	Tree base #1063
H140c	Maryland/USA/Peru	Tree base #1063	Tree base #1063	Tree base #1063	Tree base #1063
H185c	Maryland/USA/Peru	Tree base #1063	Tree base #1063	Tree base #1063	Tree base #1063
H153	Sao Paulo/Brazil	Tree base #1063	Tree base #1063	Tree base #1063	Tree base #1063
H167	Argentina	Tree base #1063	Tree base #1063	Tree base #1063	Tree base #1063
EH-383IA	Morelos/Mexico	AF495619.1	NI	NI	NI
EH-383PA	Morelos/Mexico	AF495623.1	NI	NI	NI
EH-393[a]	Oaxaca/Mexico	AF495635.1	NI	NI	NI
EH-408HA	Puebla/Mexico	AF495644.1	NI	NI	NI
EH-408PA	Puebla/Mexico	AF495647.1	NI	NI	NI
H60	Bogota/Colombia	Tree base #1063	Tree base #1063	Tree base #1063	Tree base #1063
H61	Bogota/Colombia	Tree base #1063	Tree base #1063	Tree base #1063	Tree base #1063
H62	Bogota/Colombia	Tree base #1063	Tree base #1063	Tree base #1063	Tree base #1063

H63	Bogota/Colombia	Tree base #1063	Tree base #1063	Tree base #1063	Tree base #1063
H211	Thailand	Tree base #1063	Tree base #1063	Tree base #1063	Tree base #1063
EH304	Guatemala	Tree base #1063	Tree base #1063	Tree base #1063	Tree base #1063
EH319	Mexico City/Mexico	Tree base #1063	Tree base #1063	Tree base #1063	Tree base #1063
EH332	Guatemala	Tree base #1063	Tree base #1063	Tree base #1063	Tree base #1063
EH359	Oaxaca/Mexico	Tree base #1063	Tree base #1063	Tree base #1063	Tree base #1063
EH374	Morelos/Mexico	Tree base #1063	Tree base #1063	Tree base #1063	Tree base #1063
EH375	Morelos/Mexico	AF495607.1	NI	NI	NI
EH376	Morelos/Mexico	Tree base #1063	Tree base #1063	Tree base #1063	Tree base #1063
EH379	Mexico	Tree base #1063	Tree base #1063	Tree base #1063	Tree base #1063
EH391	Morelos/Mexico	Tree base #1063	Tree base #1063	Tree base #1063	Tree base #1063
EH394	Oaxaca/Mexico	Tree base #1063	Tree base #1063	Tree base #1063	Tree base #1063
Hond4	Honduras	JQ218423.1	JQ218363.1	JQ218402.1	JQ218380.1
Hond5	Honduras	JQ218424.1	JQ218364.1	JQ218403.1	JQ218387.1
Hond15	Honduras	JQ218433.1	JQ218372.1	JQ218413.1	JQ218383.1
Hond16	Honduras	NI	JQ218373.1	JQ218414.1	JQ218390.1
Hond21	Honduras	NI	NI	JQ218417.1	JQ218384.1
Hond22	Honduras	JQ218434.1	JQ218375.1	NI	JQ218391.1
Hond23	Honduras	NI	JQ218376.1	JQ218418.1	JQ218398.1
Hond26	Honduras	JQ218436.1	JQ218378.1	JQ218420.1	JQ218392.1
H141	Indonesia	Tree base #1063	Tree base #1063	Tree base #1063	Tree base #1063
H188	Panama	Tree base #1063	Tree base #1063	Tree base #1063	Tree base #1063
2134	Texas/USA	JX443630.1	JX458485.1	JX458503.1	JX431896.1
H71	Medellin/Colombia	Tree base #1063	Tree base #1063	Tree base #1063	Tree base #1063
H73	Bogota/Colombia	Tree base #1063	Tree base #1063	Tree base #1063	Tree base #1063
H74	Medellin/Colombia	Tree base #1063	Tree base #1063	Tree base #1063	Tree base #1063
H76	Medellin/Colombia	Tree base #1063	Tree base #1063	Tree base #1063	Tree base #1063
EH46	Guerrero/Mexico	Tree base #1063	Tree base #1063	Tree base #1063	Tree base #1063
EH53	Hidalgo/Mexico	Tree base #1063	Tree base #1063	Tree base #1063	Tree base #1063
EH303	Guatemala	Tree base #1063	Tree base #1063	Tree base #1063	Tree base #1063
EH316	Guerrero/Mexico	Tree base #1063	Tree base #1063	Tree base #1063	Tree base #1063

EH333	Guatemala	Tree base #1063	Tree base #1063	Tree base #1063	Tree base #1063
EH362	Guatemala	Tree base #1063	Tree base #1063	Tree base #1063	Tree base #1063
EH363	Guatemala	Tree base #1063	Tree base #1063	Tree base #1063	Tree base #1063
EH364	Guatemala	Tree base #1063	Tree base #1063	Tree base #1063	Tree base #1063
EH372	Morelos/Mexico	Tree base #1063	Tree base #1063	Tree base #1063	Tree base #1063
EH373	Morelos/Mexico	Tree base #1063	Tree base #1063	Tree base #1063	Tree base #1063
EH377	Morelos/Mexico	Tree base #1063	Tree base #1063	Tree base #1063	Tree base #1063
EH378	Morelos/Mexico	Tree base #1063	Tree base #1063	Tree base #1063	Tree base #1063
190CLC	RS/Brazil	GU320877.1	GU320885.1	GU320987.1	GU321058.1
B670	RJ/Brazil	GU320882.1	GU320935.1	GU321035.1	GU321086.1
GO1820	Goias/Brazil	GU320864.1	GU320904.1	GU320992.1	GU321042.1
GO764	Goias/Brazil	GU320863.1	GU320905.1	GU320991.1	GU321041.1
H66	Medellin/Colombia	Tree base #1063	Tree base #1063	Tree base #1063	Tree base #1063
H69	Medellin/Colombia	Tree base #1063	Tree base #1063	Tree base #1063	Tree base #1063
Hond6	Honduras	NI	NI	JQ218404.1	JQ218394.1
H59	Bogota/Colombia	Tree base #1063	Tree base #1063	Tree base #1063	Tree base #1063
H68	Medellin/Colombia	Tree base #1063	Tree base #1063	Tree base #1063	Tree base #1063
H70	Medellin/Colombia	Tree base #1063	Tree base #1063	Tree base #1063	Tree base #1063
H75	Medellin/Colombia	Tree base #1063	Tree base #1063	Tree base #1063	Tree base #1063
H85	Argentina	Tree base #1063	Tree base #1063	Tree base #1063	Tree base #1063
H162	Argentina	Tree base #1063	Tree base #1063	Tree base #1063	Tree base #1063
H163	Argentina	Tree base #1063	Tree base #1063	Tree base #1063	Tree base #1063
H164	Argentina	Tree base #1063	Tree base #1063	Tree base #1063	Tree base #1063
H166	Argentina	Tree base #1063	Tree base #1063	Tree base #1063	Tree base #1063
H168	Argentina	Tree base #1063	Tree base #1063	Tree base #1063	Tree base #1063
H169	Argentina	Tree base #1063	Tree base #1063	Tree base #1063	Tree base #1063
H171	Argentina	Tree base #1063	Tree base #1063	Tree base #1063	Tree base #1063
H172	Argentina	Tree base #1063	Tree base #1063	Tree base #1063	Tree base #1063
MS53	MS/Brazil	GU320847.1	GU320934.1	GU321036.1	GU321076.1
157CS	RS/Brazil	GU320875.1	GU320887.1	GU321009.1	GU321056.1
177CS	RS/Brazil	GU320884.1	GU320933.1	GU321037.1	GU321087.1

187LCT	RS/Brazil	GU320876.1	GU320886.1	GU321010.1	GU321057.1
184PRS	RS/Brazil	GU320883.1	GU320932.1	GU321011.1	GU321088.1
ES55	Espirito Santo/Brazil	GU320868.1	GU320900.1	GU320996.1	GU321046.1
ES56	Espirito Santo/Brazil	GU320869.1	GU320899.1	GU320997.1	GU321047.1
ES60	Espirito Santo/Brazil	GU320870.1	GU320898.1	GU320998.1	GU321048.1
EH-384IA	Oaxaca/Mexico	AF495627.1	NI	NI	NI
EH-384PA	Oaxaca/Mexico	AF495631.1	NI	NI	NI
EH315	Guerrero/Mexico	Tree base #1063	Tree base #1063	Tree base #1063	Tree base #1063
H81	Panamá	Tree base #1063	Tree base #1063	Tree base #1063	Tree base #1063
H82	Panamá	Tree base #1063	Tree base #1063	Tree base #1063	Tree base #1063
H83	Panamá	Tree base #1063	Tree base #1063	Tree base #1063	Tree base #1063
3356	Rio de Janeiro/Brazil	GU320879.1	GU320909.1	GU321033.1	GU321059.1
3688	Rio de Janeiro/Brazil	GU320834.1	GU320929.1	GU321025.1	GU321062.1
4334	Rio de Janeiro/Brazil	GU320835.1	GU320912.1	GU321026.1	GU321063.1
6406	Rio de Janeiro/Brazil	GU320837.1	GU320914.1	GU321034.1	GU321065.1
9414	Rio de Janeiro/Brazil	GU320873.1	GU320892.1	GU321004.1	GU321054.1
AC02	RJ/Brazil	GU320858.1	GU320916.1	GU321013.1	GU321069.1
AC05	RJ/Brazil	GU320859.1	GU320917.1	GU321021.1	GU321070.1
CAO4	RJ/Brazil	GU320844.1	GU320919.1	GU321022.1	GU321072.1
EP02	Rio de Janeiro/Brazil	GU320878.1	GU320920.1	GU321015.1	GU321073.1
H150	Sao Paulo/Brazil	Tree base #1063	Tree base #1063	Tree base #1063	Tree base #1063
H152	Sao Paulo/Brazil	Tree base #1063	Tree base #1063	Tree base #1063	Tree base #1063
H155	Sao Paulo/Brazil	Tree base #1063	Tree base #1063	Tree base #1063	Tree base #1063
H156	Sao Paulo/Brazil	Tree base #1063	Tree base #1063	Tree base #1063	Tree base #1063
H198	Rio de Janeiro/Brazil	Tree base #1063	Tree base #1063	Tree base #1063	Tree base #1063
H199	Rio de Janeiro/Brazil	Tree base #1063	Tree base #1063	Tree base #1063	Tree base #1063
H200	Rio de Janeiro/Brazil	Tree base #1063	Tree base #1063	Tree base #1063	Tree base #1063
H201	Rio de Janeiro/Brazil	Tree base #1063	Tree base #1063	Tree base #1063	Tree base #1063
H202	Rio de Janeiro/Brazil	Tree base #1063	Tree base #1063	Tree base #1063	Tree base #1063
H203	Rio de Janeiro/Brazil	Tree base #1063	Tree base #1063	Tree base #1063	Tree base #1063
IGS19	Rio de Janeiro/Brazil	GU320855.1	GU320895.1	GU321001.1	GU321051.1

IGS4/5	Rio de Janeiro/Brazil	GU320845.1	GU320922.1	GU321029.1	GU321074.1
IT04	Rio de Janeiro/Brazil	GU320846.1	GU321074.1	GU321020.1	GU321075.1
RS01	Rio de Janeiro/Brazil	GU320853.1	GU320921.1	GU321032.1	GU321080.1
RS09	Rio de Janeiro/Brazil	GU320854.1	GU320894.1	GU321002.1	GU321052.1
3416	Rio de Janeiro/Brazil	GU320880.1	GU320910.1	GU321023.1	GU321060.1
3612	Rio de Janeiro/Brazil	GU320881.1	GU320911.1	GU321024.1	GU321061.1
4631	Rio de Janeiro/Brazil	GU320836.1	GU320913.1	GU321027.1	GU321064.1
9291	Rio de Janeiro/Brazil	GU320874.1	GU320891.1	GU321005.1	GU321055.1
CADAM	RJ/Brazil	GU320843.1	GU320918.1	GU321014.1	GU321071.1
6503	Rio de Janeiro/Brazil	GU320838.1	GU320930.1	GU321028.1	GU321066.1
RPS51	Rio de Janeiro/Brazil	GU320848.1	GU320924.1	GU321030.1	GU321077.1
RPS86	Rio de Janeiro/Brazil	GU320856.1	GU320925.1	GU321031.1	GU321078.1
TI01	Rio de Janeiro/Brazil	GU320850.1	GU320926.1	GU321017.1	GU321081.1
TI05	Rio de Janeiro/Brazil	GU320851.1	GU320927.1	GU321018.1	GU321082.1
TI14	Rio de Janeiro/Brazil	GU320852.1	GU320928.1	GU321019.1	GU321083.1

Fig S1

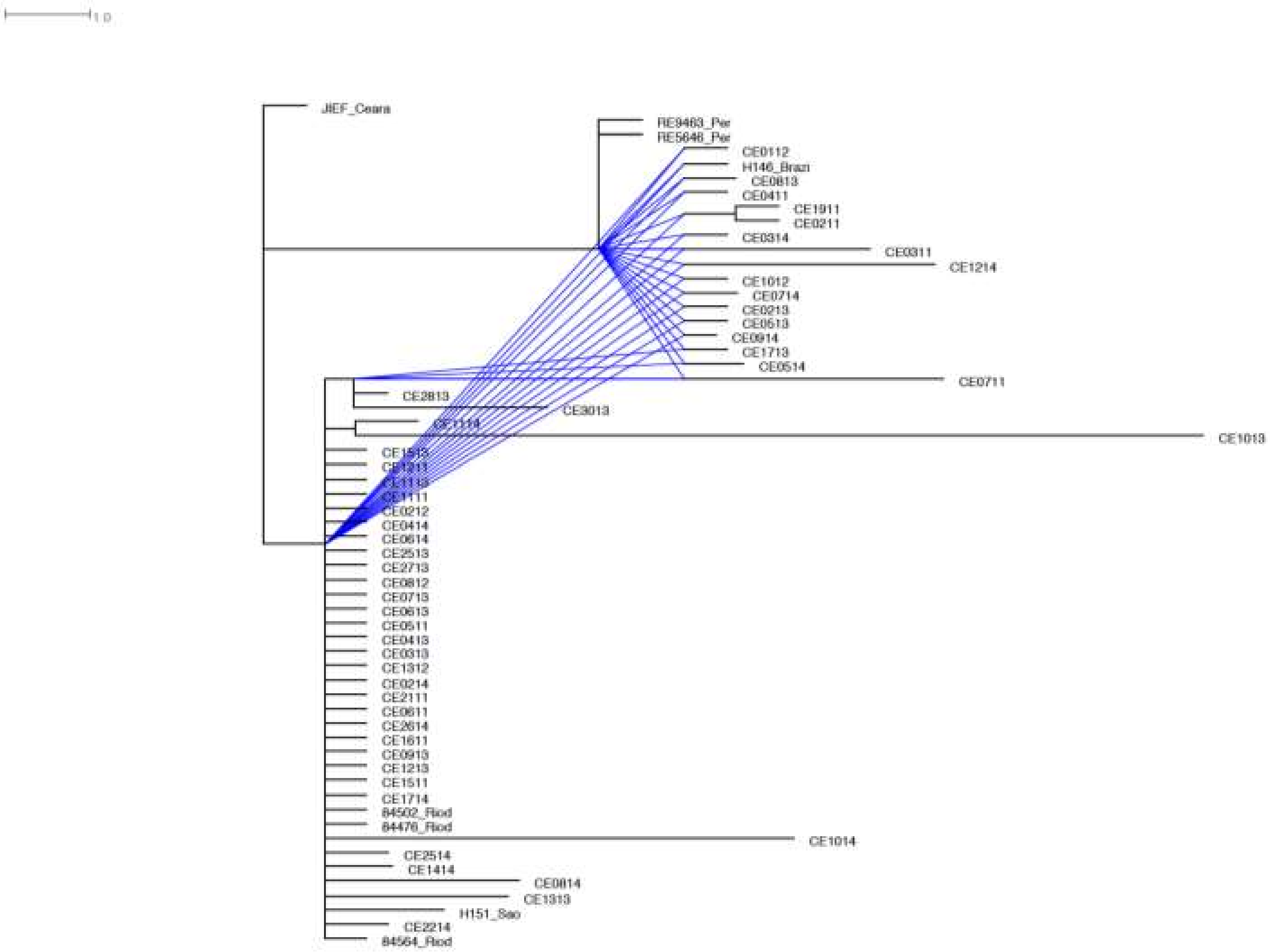

Figure S1 – Cluster network analysis representing multiple phylogenetic hypotheses simultaneously from individuals belonging to the Northeast BR1 and Northeast BR2 clades.

Fig S2

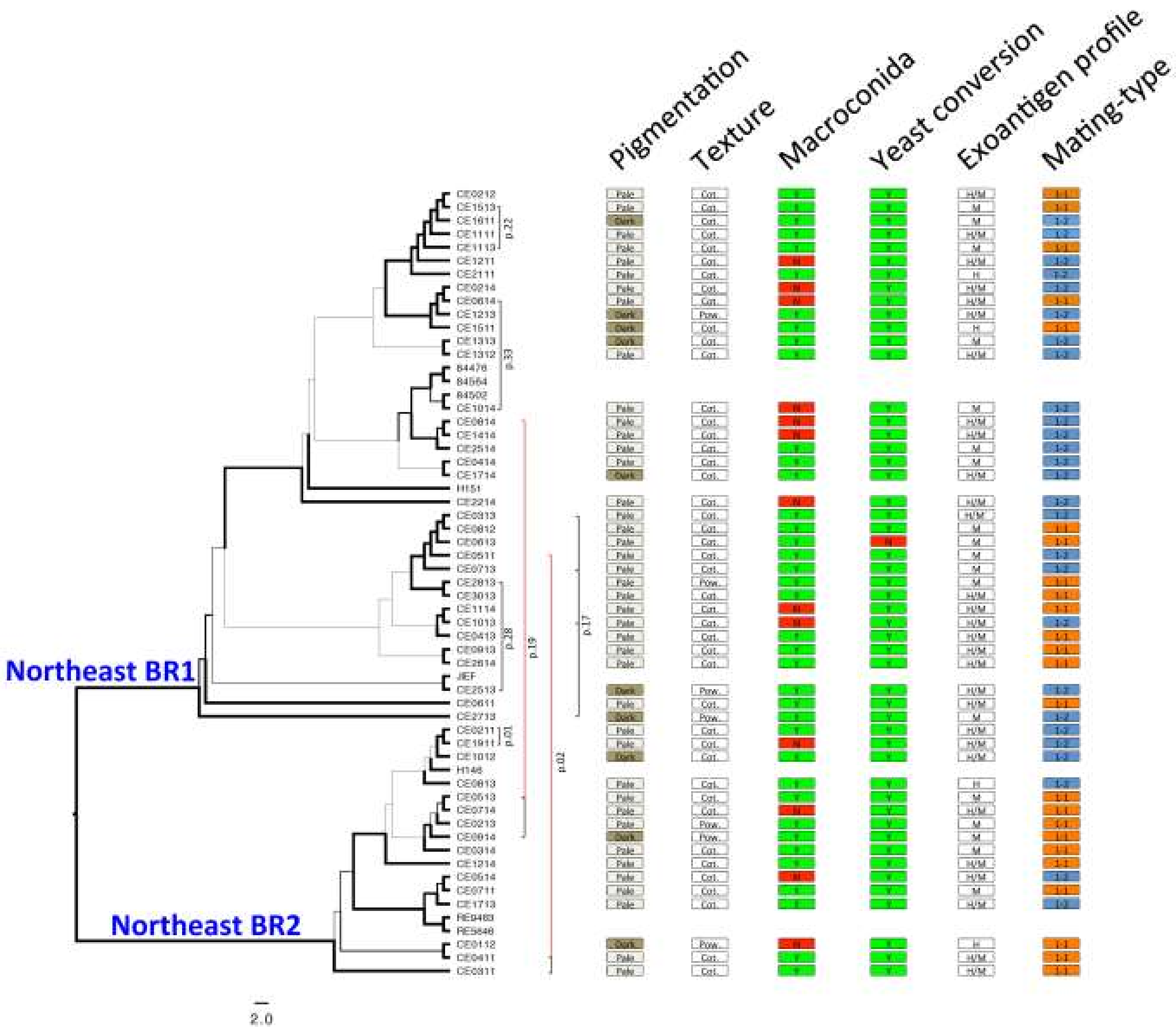

Figure S2 – Maximum Likelihood analysis of *H. capsulatum* strains recovered from the Northeast region of Brazil, associate phenotypes and patient source for multiple infecting lineages.

Colony pigmentation and texture, production of micro and macroconidia, yeast conversion, exoantigen production and mating type were accessed and are displayed next to each taxon. Multiple strains recovered from a single patient are connected by brackets along the phylogenetic tree.

Fig S3

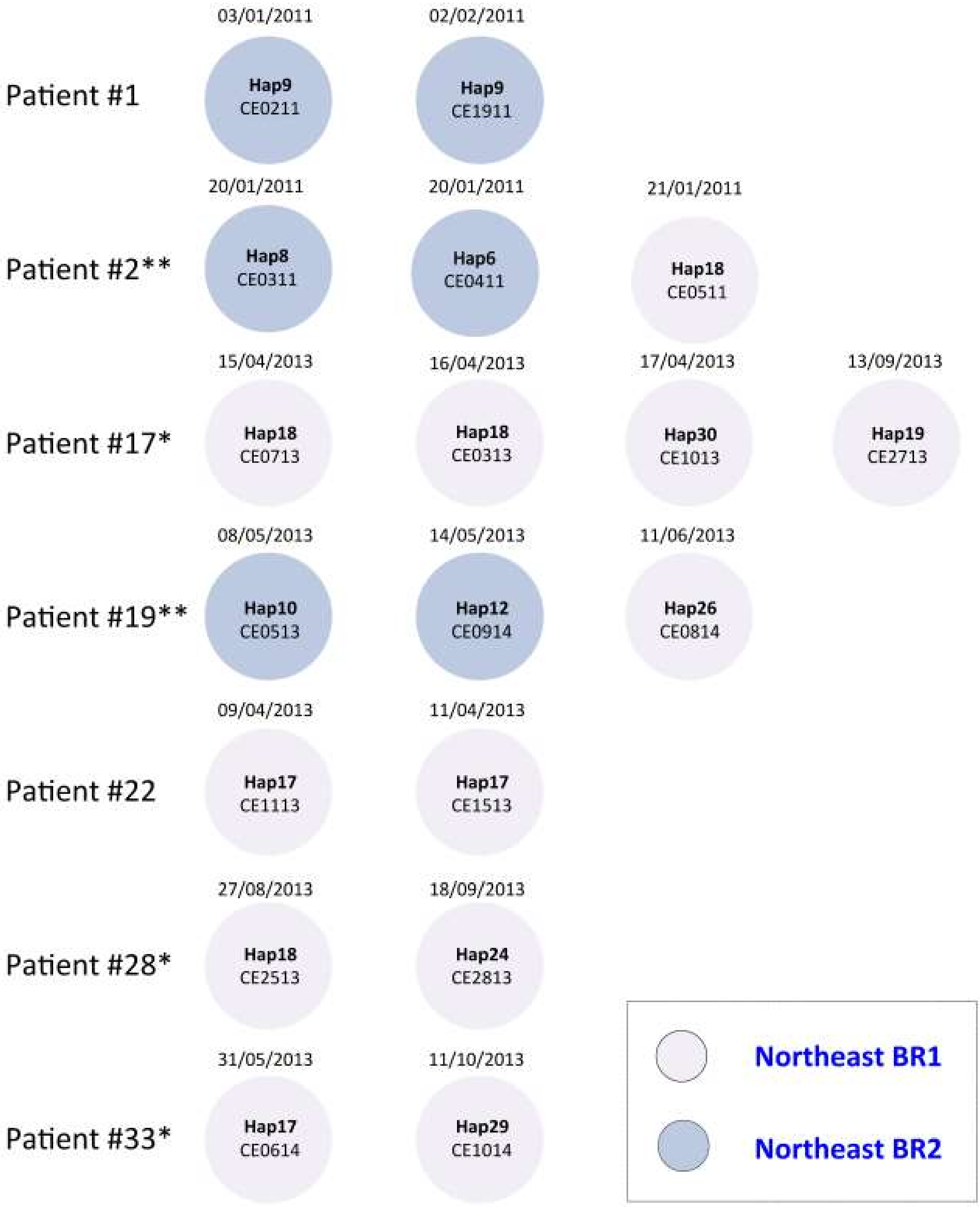

Figure S3 – Course of *H. capsulatum* isolation from sampled patients.
Patients that had two or more isolations in different days are displayed and the genotype of each isolation are plotted in each circle that represent a single event of culture isolation.
